# Comprehensive registry of esophageal cancer in Japan, 2015

**DOI:** 10.1007/s10388-022-00950-5

**Published:** 2022-09-24

**Authors:** Masayuki Watanabe, Yasushi Toh, Ryu Ishihara, Koji Kono, Hisahiro Matsubara, Tatsuya Miyazaki, Masaru Morita, Kentaro Murakami, Kei Muro, Hodaka Numasaki, Tsuneo Oyama, Hiroshi Saeki, Koji Tanaka, Takahiro Tsushima, Masaki Ueno, Takashi Uno, Toshiyuki Yoshio, Shiyori Usune, Arata Takahashi, Hiroaki Miyata, The Registration Committee for Esophageal Cancer of the Japan Esophageal Society

**Affiliations:** 1 Department of Gastroenterological Surgery, Cancer Institute Hospital of Japanese Foundation for Cancer Research, 3-8-31 Ariake, Koto-ku, Tokyo, 135-8550 Japan; 2 Department of Gastroenterological Surgery, National Hospital Organization Kyushu Cancer Center, 3-1-1 Notame, Minami-ku, Fukuoka, 811-1395 Japan; 3 Department of Gastrointestinal Oncology, Osaka International Cancer Institute, 3-1-69 Otemae, Chuo-ku, Osaka, 541-8567 Japan; 4 Department of Gastrointestinal Tract Surgery, Fukushima Medical University School of Medicine, 1 Hikarigaoka, Fukushima, 960-1295 Japan; 5 Department of Frontier Surgery, Graduate School of Medicine, Chiba University, 1-8-1 Inohana, Chuo-ku, Chiba, 260-8670 Japan; 6 Department of Surgery, Japanese Red Cross Maebashi Hospital, 389-1 Asakura-machi, Maebashi, Gunma 371-0811 Japan; 7 Department of Clinical Oncology, Aichi Cancer Center Hospital, 1-1 Kanokoden, Chikusa-ku, Nagoya, 464-8681 Japan; 8 Department of Medical Physics and Engineering, Graduate School of Medicine, Osaka University, 2-2 Yamadaoka, Suita, Osaka 565-0871 Japan; 9 Department of Endoscopy, Saku Central Hospital Advanced Care Center, 3400-28 Nakagomi, Saku, Nagano 385-0051 Japan; 10 Department of General Surgical Science, Graduate School of Medicine, Gunma University, 3-39-22 Showa-machi, Maebashi, Gunma 371-8511 Japan; 11 Department Gastroenterological Surgery, Graduate School of Medicine, Osaka University, 2-2 Yamadaoka, Suita, Osaka 565-0871 Japan; 12 Division of Gastroenterological Oncology, Shizuoka Cancer Center, 1007 Shimonagakubo, Nagaizumi-cho, Sunto-gun, Shizuoka, 411-8777 Japan; 13 Department of Gastroenterological Surgery, Toranomon Hospital, 2-2-2 Toranomon, Minato-ku, Tokyo, 105-8470 Japan; 14 Department of Diagnostic Radiology and Radiation Oncology, Graduate School of Medicine, Chiba University, 1-8-1 Inohana, Chuo-ku, Chiba, 260-8670 Japan; 15 Department of Upper Gastrointestinal Medicine, Cancer Institute Hospital of Japanese Foundation for Cancer Research, 3-8-31 Ariake, Koto-ku, Tokyo, 135-8550 Japan; 16 Department of Healthcare Quality Assessment, Graduate School of Medicine, The University of Tokyo, 7-3-1 Hongo, Bunkyo-ku, Tokyo, 113-8655 Japan

**Keywords:** Esophageal cancer, Esophagectomy, Endoscopic resection, Chemotherapy, Chemoradiotherapy, Cancer registry

## Abstract

**Background:**

The registration committee for esophageal cancer in the Japan Esophageal Society (JES) has collected the patients' characteristics, treatment, and outcomes of patients who underwent any treatment during 2015 in Japan.

**Methods:**

We analyzed patients' data who had visited the participating hospitals in 2015. We collected the data using the National Clinical Database with a web-based data collection system. We used the Japanese Classification of Esophageal Cancer 10^th^ edition by JES and the TNM classification by the Union of International Cancer Control (UICC) for cancer staging.

**Results:**

A total of 9368 cases were registered from 355 institutions in Japan. Squamous cell carcinoma and adenocarcinoma accounted for 86.7% and 7.4%, respectively. The 5-year survival rates of patients treated by endoscopic resection, concurrent chemoradiotherapy, radiotherapy alone, and esophagectomy were 87.2%, 33.5%, 24.2%, and 59.9%, respectively. Esophagectomy was performed in 5172 cases. Minimally invasive approaches were selected for 60.6%, and 54.4% underwent thoracoscopic esophagectomy. The operative mortality (within 30 days after surgery) was 0.79% and the hospital mortality was 2.3%. The survival curves showed an excellent discriminatory ability both in the clinical and pathologic stages by the JES system. The survival of pStage IV was better than IIIC in the UICC system because pStage IV included the patients with supraclavicular lymph node metastasis (M1 LYM).

**Conclusion:**

We hope this report improves all aspects of diagnosing and treating esophageal cancer in Japan.

## Preface 2015

We sincerely appreciate the outstanding contributions of many physicians in the registry of esophageal cancer cases. The Comprehensive Registry of Esophageal Cancer in Japan, 2015, was published here. In 2019, the data collection method was changed from an electronic submission to a web-based data collection using the National Clinical Database (NCD). Personal information was replaced with individual management codes inside each institute, and the NCD collected only anonymized information. The registry complies with the Act for the Protection of Personal Information.

We briefly summarized the Comprehensive Registry of Esophageal Cancer in Japan, 2015. According to the subject year, we used the Japanese Classification of Esophageal Cancer 10th by the Japan Esophageal Society (JES) [1] and the Union of International Cancer Control (UICC) TNM Classification 7th [2] for cancer staging. A total of 9368 cases were registered from 355 institutions in Japan. Tumor locations were cervical in 4.6%, upper thoracic in 12.1%, middle thoracic in 46.0%, lower thoracic in 27.9%, and esophagogastric junction in 8.5%. Superficial carcinomas (Tis, T1a, T1b) were 38.2%. As for the histologic type of biopsy specimens, squamous cell carcinoma and adenocarcinoma accounted for 86.7% and 7.4%, respectively. Regarding clinical results, the 5-year survival rates of patients treated using endoscopic resection, concurrent chemoradiotherapy, radiotherapy alone, and esophagectomy were 87.2%, 33.5%, 24.2%, and 59.9%, respectively. The endoscopic submucosal dissection accounted for 92.9% of endoscopic resection. Esophagectomy was performed in 5172 cases. Minimally invasive approaches were selected for 60.6%, and 54.4% underwent thoracoscopic esophagectomy. The operative mortality (within 30 days after surgery) was 0.79%, and the hospital mortality was 2.3%. The N-grade significantly differed between the JES and the UICC systems; based on the location of metastatic lymph nodes in the JES system and the number of metastatic nodes in the UICC system. However, the N-grades effectively estimated the survival in both the JES and the UICC systems. The survival curves showed an excellent discriminatory ability both in the clinical and pathologic stages by the JES system. In contrast, in the UICC system, the survival of cStage IIB was identical to IB and better than IIA, and the survival curves were similar between cStage IIIC and IV. Also, the survival curve of pStage IIB was superior to IIA, and the survival of pStage IV was better than IIIC. pStage IV in the UICC system included the patients with supraclavicular lymph node metastasis (M1 LYM), which is possibly the reason for the better prognosis of pStage IV than IIIC.

We hope that this Comprehensive Registry of Esophageal Cancer in Japan 2015 will help improve all aspects of the diagnosis and treatment of esophageal cancer in Japan.

## Contents

### I. Clinical factors of esophageal cancer patients treated in 2015

#### 1. Institution-registered cases in 2015

#### 2. Patient background

#### Table 1 Age and gender

**Table 1:** Age and gender

Age	Male	Female	Cases (%)
≤ 29	14	5	19 (0.2%)
30–39	17	11	28 (0.3%)
40–49	171	93	264 (2.8%)
50–59	946	227	1173 (12.5%)
60–69	2928	559	3487 (37.2%)
70–79	2954	502	3456 (36.9%)
80–89	713	185	898 (9.6%)
90 ≤	25	18	43 (0.5%)
Total	7768	1600	9368

#### Table 2 Performed treatment

**Table 2:** Performed treatment

Treatments	Cases (%)
Surgery	5354 (57.2%)
Esophagectomy	5172 (55.2%)
Palliative surgery	182 (1.9%)
Chemotherapy and/or Radiotherapy	5119 (54.6%)
Chemoradiotherapy	1207 (12.9%)
Radiotherapy alone	330 (3.5%)
Chemotherapy alone	450 (4.8%)
Palliative radiation	112 (1.2%)
Others	3020 (32.2%)
Endoscopic treatment	1709 (18.2%)

#### Table 3 Tumor location

**Table 3:** Tumor location

Location of tumor	Endoscopic treatment (%)	Surgery		Chemotherapy and/or radiotherapy					Total (%)
		Esophagectomy (%)	Palliative surgery (%)	CRT (%)	RT alone (%)	Chemotherapy alone (%)	Palliative radiotherapy (%)	Others (%)	
Cervical	39 (2.3%)	155 (3.0%)	11 (6.0%)	147 (12.2%)	32 (9.7%)	22 (4.9%)	6 (5.4%)	107 (3.5%)	435 (4.6%)
Upper thoracic	171 (10.0%)	581 (11.2%)	24 (13.2%)	220 (18.2%)	56 (17.0%)	39 (8.7%)	9 (8.0%)	373 (12.4%)	1131 (12.1%)
Middle thoracic	903 (52.8%)	2305 (44.6%)	99 (54.4%)	555 (46.0%)	142 (43.0%)	177 (39.3%)	57 (50.9%)	1364 (45.2%)	4308 (46.0%)
Lower thoracic	430 (25.2%)	1585 (30.6%)	39 (21.4%)	252 (20.9%)	76 (23.0%)	171 (38.0%)	33 (29.5%)	946 (31.3%)	2609 (27.9%)
EG	110 (6.4%)	393 (7.6%)	2 (1.1%)	20 (1.7%)	8 (2.4%)	18 (4.0%)	6 (5.4%)	166 (5.5%)	562 (6.0%)
E = G	30 (1.8%)	75 (1.5%)	3 (1.6%)	3 (0.2%)	0 (0.0%)	5 (1.1%)	1 (0.9%)	26 (0.9%)	118 (1.3%)
GE	6 (0.4%)	71 (1.4%)	3 (1.6%)	3 (0.2%)	1 (0.3%)	10 (2.2%)	0 (0.0%)	34 (1.1%)	110 (1.2%)
unknown	20 (1.2%)	7 (0.1%)	1 (0.5%)	7 (0.6%)	15 (4.5%)	8 (1.8%)	0 (0.0%)	4 (0.1%)	95 (1.0%)
Total	1709	5172	182	1207	330	450	112	3020	9368

*E* esophageal, *G* gastric

#### Table 4 Histologic types of biopsy specimens

**Table 4:** Histologic type of biopsy specimens

Histologic types	Endoscopic treatment (%)	Surgery		Chemotherapy and/or radiotherapy					Total (%)
		Esophagectomy (%)	Palliative surgery (%)	CRT (%)	RT alone (%)	Chemotherapy alone (%)	Palliative RT (%)	Others (%)	
Squamous cell carcinoma	1405 (82.2%)	4524 (87.5%)	165 (90.7%)	1152 (95.4%)	307 (93.0%)	360 (80.0%)	103 (92.0%)	2730 (90.4%)	8123 (86.7%)
Squamous cell carcinoma	1065 (62.3%)	2486 (48.1%)	114 (62.6%)	764 (63.3%)	205 (62.1%)	221 (49.1%)	59 (52.7%)	1566 (51.9%)	5041 (53.8%)
Well differentiated	136 (8.0%)	421 (8.1%)	11 (6.0%)	60 (5.0%)	26 (7.9%)	32 (7.1%)	5 (4.5%)	227 (7.5%)	687 (7.3%)
Moderately differentiated	179 (10.5%)	1231 (23.8%)	28 (15.4%)	223 (18.5%)	53 (16.1%)	69 (15.3%)	28 (25.0%)	710 (23.5%)	1783 (19.0%)
Poorly differentiated	25 (1.5%)	386 (7.5%)	12 (6.6%)	105 (8.7%)	23 (7.0%)	38 (8.4%)	11 (9.8%)	227 (7.5%)	612 (6.5%)
Adenocarcinoma	56 (3.3%)	358 (6.9%)	8 (4.4%)	17 (1.4%)	3 (0.9%)	41 (9.1%)	2 (1.8%)	190 (6.3%)	507 (5.4%)
Barrett's carcinoma	56 (3.3%)	116 (2.2%)	1 (0.5%)	0 (0.0%)	1 (0.3%)	10 (2.2%)	2 (1.8%)	17 (0.6%)	187 (2.0%)
Adenosquamous carcinoma	3 (0.2%)	16 (0.3%)	0 (0.0%)	3 (0.2%)	1 (0.3%)	3 (0.7%)	0 (0.0%)	10 (0.3%)	23 (0.2%)
Mucoepidermoid carcinoma	0 (0.0%)	3 (0.1%)	0 (0.0%)	0 (0.0%)	0 (0.0%)	0 (0.0%)	0 (0.0%)	3 (0.1%)	4 (0.0%)
Basaloid carcinoma	4 (0.2%)	43 (0.8%)	1 (0.5%)	5 (0.4%)	0 (0.0%)	4 (0.9%)	0 (0.0%)	15 (0.5%)	56 (0.6%)
Neuroendocrine tumor	1 (0.1%)	0 (0.0%)	0 (0.0%)	0 (0.0%)	0 (0.0%)	2 (0.4%)	0 (0.0%)	0 (0.0%)	3 (0.0%)
Neuroendocrine carcinoma	1 (0.1%)	19 (0.4%)	1 (0.5%)	9 (0.7%)	1 (0.3%)	10 (2.2%)	1 (0.9%)	16 (0.5%)	47 (0.5%)
Undifferentiated carcinoma	1 (0.1%)	5 (0.1%)	0 (0.0%)	3 (0.2%)	0 (0.0%)	1 (0.2%)	0 (0.0%)	3 (0.1%)	10 (0.1%)
Malignant melanoma	2 (0.1%)	20 (0.4%)	0 (0.0%)	0 (0.0%)	0 (0.0%)	3 (0.7%)	0 (0.0%)	5 (0.2%)	27 (0.3%)
Carcinosarcoma	0 (0.0%)	15 (0.3%)	1 (0.5%)	0 (0.0%)	0 (0.0%)	0 (0.0%)	1 (0.9%)	5 (0.2%)	18 (0.2%)
GIST	1 (0.1%)	4 (0.1%)	0 (0.0%)	0 (0.0%)	0 (0.0%)	0 (0.0%)	0 (0.0%)	1 (0.0%)	8 (0.1%)
Adenoid cyctic carcinoma	1 (0.1%)	4 (0.1%)	0 (0.0%)	1 (0.1%)	0 (0.0%)	0 (0.0%)	0 (0.0%)	1 (0.0%)	5 (0.1%)
Nonepithelial tumors	2 (0.1%)	3 (0.1%)	0 (0.0%)	0 (0.0%)	0 (0.0%)	0 (0.0%)	0 (0.0%)	1 (0.0%)	6 (0.1%)
Other epithelial tumors	23 (1.3%)	6 (0.1%)	0 (0.0%)	1 (0.1%)	0 (0.0%)	2 (0.4%)	0 (0.0%)	3 (0.1%)	34 (0.4%)
Other tumors	50 (2.9%)	7 (0.1%)	0 (0.0%)	2 (0.2%)	3 (0.9%)	2 (0.4%)	1 (0.9%)	3 (0.1%)	77 (0.8%)
Unknown	103 (6.0%)	29 (0.6%)	5 (2.7%)	14 (1.2%)	14 (4.2%)	12 (2.7%)	2 (1.8%)	17 (0.6%)	233 (2.5%)
Total	1709	5172	182	1207	330	450	112	3020	9368

*GIST* gastrointestinal stromal tumor

#### Table 5 Depth of tumor invasion, cT (UICC TNM 7th)

**Table 5:** Depth of tumor invasion, cT (UICC TNM 7^th^)

Clinical T	Endoscopic treatment (%)	Surgery		Chemotherapy and/or radiotherapy					Total (%)
		Esophagectomy (%)	Palliative surgery (%)	CRT (%)	RT alone (%)	Chemotherapy alone (%)	Palliative RT (%)	Others (%)	
cT0	12 (0.7%)	4 (0.1%)	0 (0.0%)	0 (0.0%)	0 (0.0%)	0 (0.0%)	1 (0.9%)	0 (0.0%)	17 (0.2%)
cT1a	1332 (77.9%)	240 (4.6%)	1 (0.5%)	46 (3.8%)	16 (4.8%)	8 (1.8%)	4 (3.6%)	70 (2.3%)	1698 (18.1%)
cT1b	242 (14.2%)	1392 (26.9%)	5 (2.7%)	177 (14.7%)	66 (20.0%)	31 (6.9%)	4 (3.6%)	362 (12.0%)	1880 (20.1%)
cT2	20 (1.2%)	857 (16.6%)	6 (3.3%)	107 (8.9%)	48 (14.5%)	50 (11.1%)	14 (12.5%)	525 (17.4%)	1123 (12.0%)
cT3	54 (3.2%)	2315 (44.8%)	78 (42.9%)	435 (36.0%)	114 (34.5%)	217 (48.2%)	62 (55.4%)	1702 (56.4%)	3294 (35.2%)
cT4a	9 (0.5%)	174 (3.4%)	15 (8.2%)	120 (9.9%)	21 (6.4%)	44 (9.8%)	2 (1.8%)	126 (4.2%)	411 (4.4%)
cT4b	23 (1.3%)	172 (3.3%)	73 (40.1%)	308 (25.5%)	49 (14.8%)	77 (17.1%)	23 (20.5%)	226 (7.5%)	786 (8.4%)
cTX	17 (1.0%)	18 (0.3%)	4 (2.2%)	14 (1.2%)	16 (4.8%)	23 (5.1%)	2 (1.8%)	9 (0.3%)	159 (1.7%)
Total	1709	5172	182	1207	330	450	112	3020	9368

#### Table 6 Lymph node metastasis, cN (UICC TNM 7th)

**Table 6:** Lymph node metastasis, cN (UICC TNM 7^th^)

Clinical N	Endoscopic treatment (%)	Surgery		Chemotherapy and/or radiotherapy					Total (%)
		Esophagectomy (%)	Palliative surgery (%)	CRT (%)	RT alone (%)	Chemotherapy alone (%)	Palliative RT (%)	Others (%)	
cN0	1612 (94.3%)	2365 (45.7%)	19 (10.4%)	327 (27.1%)	128 (38.8%)	69 (15.3%)	22 (19.6%)	844 (27.9%)	4621 (49.3%)
cN1	54 (3.2%)	1752 (33.9%)	57 (31.3%)	389 (32.2%)	119 (36.1%)	139 (30.9%)	30 (26.8%)	1290 (42.7%)	2602 (27.8%)
cN2	31 (1.8%)	901 (17.4%)	76 (41.8%)	346 (28.7%)	63 (19.1%)	144 (32.0%)	37 (33.0%)	745 (24.7%)	1642 (17.5%)
cN3	12 (0.7%)	154 (3.0%)	30 (16.5%)	145 (12.0%)	20 (6.1%)	98 (21.8%)	23 (20.5%)	141 (4.7%)	503 (5.4%)
cNX	0 (0.0%)	0 (0.0%)	0 (0.0%)	0 (0.0%)	0 (0.0%)	0 (0.0%)	0 (0.0%)	0 (0.0%)	0 (0.0%)
Total	1709	5172	182	1207	330	450	112	3020	9368

#### Table 7 Distant metastasis, cM (UICC TNM 7th)

**Table 7:** Distant metastasis, cM (UICC TNM 7th)

Clinical M	Endoscopic treatment (%)	Surgery		Chemotherapy and/or radiotherapy					Total (%)
		Esophagectomy (%)	Palliative surgery (%)	CRT (%)	RT alone (%)	Chemotherapy alone (%)	Palliative RT (%)	Others (%)	
cM0	1680 (98.3%)	4976 (96.2%)	133 (73.1%)	897 (74.3%)	266 (80.6%)	218 (48.4%)	86 (76.8%)	2814 (93.2%)	8370 (89.3%)
cM1	29 (1.7%)	196 (3.8%)	49 (26.9%)	310 (25.7%)	64 (19.4%)	232 (51.6%)	26 (23.2%)	206 (6.8%)	998 (10.7%)
Total	1709	5172	182	1207	330	450	112	3020	9368

#### Table 8 Clinical stage (UICC TNM 7th)

**Table 8:** Clinical Stage (UICC TNM 7th)

Clinical stage	Endoscopic treatment (%)	Surgery		Chemotherapy and/or radiotherapy					Total (%)
		Esophagectomy (%)	Palliative surgery (%)	CRT (%)	RT alone (%)	Chemotherapy alone (%)	Palliative RT (%)	Others (%)	
Stage IA	1557 (91.1%)	1291 (25.0%)	5 (2.7%)	175 (14.5%)	70 (21.2%)	12 (2.7%)	4 (3.6%)	234 (7.7%)	3126 (33.4%)
Stage IB	10 (0.6%)	448 (8.7%)	3 (1.6%)	36 (3.0%)	15 (4.5%)	17 (3.8%)	8 (7.1%)	235 (7.8%)	548 (5.8%)
Stage IIA	6 (0.4%)	537 (10.4%)	5 (2.7%)	62 (5.1%)	25 (7.6%)	17 (3.8%)	7 (6.3%)	324 (10.7%)	661 (7.1%)
Stage IIB	19 (1.1%)	568 (11.0%)	2 (1.1%)	63 (5.2%)	31 (9.4%)	22 (4.9%)	3 (2.7%)	353 (11.7%)	715 (7.6%)
Stage IIIA	21 (1.2%)	1146 (22.2%)	30 (16.5%)	136 (11.3%)	50 (15.2%)	51 (11.3%)	20 (17.9%)	858 (28.4%)	1454 (15.5%)
Stage IIIB	9 (0.5%)	575 (11.1%)	20 (11.0%)	96 (8.0%)	18 (5.5%)	31 (6.9%)	16 (14.3%)	434 (14.4%)	758 (8.1%)
Stage IIIC	30 (1.8%)	391 (7.6%)	68 (37.4%)	322 (26.7%)	49 (14.8%)	61 (13.6%)	26 (23.2%)	370 (12.3%)	992 (10.6%)
Stage IV	29 (1.7%)	196 (3.8%)	49 (26.9%)	310 (25.7%)	64 (19.4%)	232 (51.6%)	26 (23.2%)	206 (6.8%)	998 (10.7%)
Unknown	28 (1.6%)	20 (0.4%)	0 (0.0%)	7 (0.6%)	8 (2.4%)	7 (1.6%)	2 (1.8%)	6 (0.2%)	116 (1.2%)
Total	1709	5172	182	1207	330	450	112	3020	9368

### II. Results of endoscopically treated patients in 2015

#### Table 9 Details of endoscopic treatment for curative intent

**Table 9:** Details of endoscopic treatment for curative intent

Treatment details	Cases (%)
EMR	114 (6.8%)
EMR + YAG laser	1 (0.1%)
EMR + MCT or RFA	0 (0.0%)
ESD	1537 (91.2%)
ESD + EMR	14 (0.8%)
ESD + PDT	0 (0.0%)
ESD + YAG laser	1 (0.1%)
PDT	5 (0.3%)
YAG laser	14 (0.8%)
Total	1686

*EMR* endoscopic mucosal resection, *PDT* photodynamic therapy, *YAG* yttrium aluminum garnet, *MCT* microwave coagulation therapy, *ESD* endoscopic submucosal dissection

#### Table 10 Complications of EMR/ESD

**Table 10:** Complications of EMR/ESD

Complications of EMR/ESD	Cases (%)
None	1599 (96.0%)
Perforation	15 (0.9%)
Bleeding	2 (0.1%)
Mediastinitis	2 (0.1%)
Stenosis	45 (2.7%)
Others	0 (0.0%)
Unknown	2 (0.1%)
Total	1665

#### Table 11 Pathologic depth of tumor invasion of EMR/ESD specimens

**Table 11:** Pathologic depth of tumor invasion of EMR/ESD specimens

Pathological depth of tumor invasion (pT)	Cases (%)
pT0	34 (2.0%)
pT1a	1315 (78.8%)
pT1b	291 (17.4%)
pT2	6 (0.4%)
pT3	0 (0.0%)
pTX	23 (1.4%)
Total	1669

#### Figure 1 Survival of patients treated with EMR/ESD

**Fig. 1:**
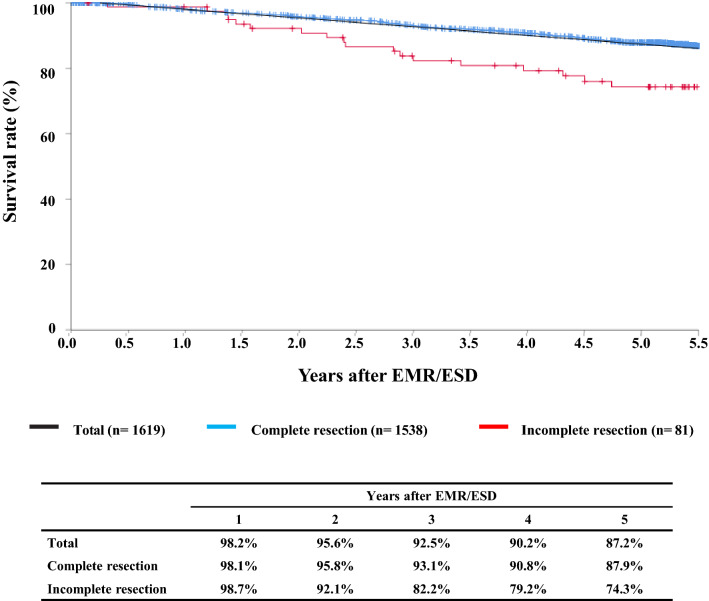
Survival of patients treated with EMR/ESD

#### Figure 2 Survival of patients treated with EM/ESD according to the pathological depth of tumor invasion, pT (JES 10th)

**Fig. 2:**
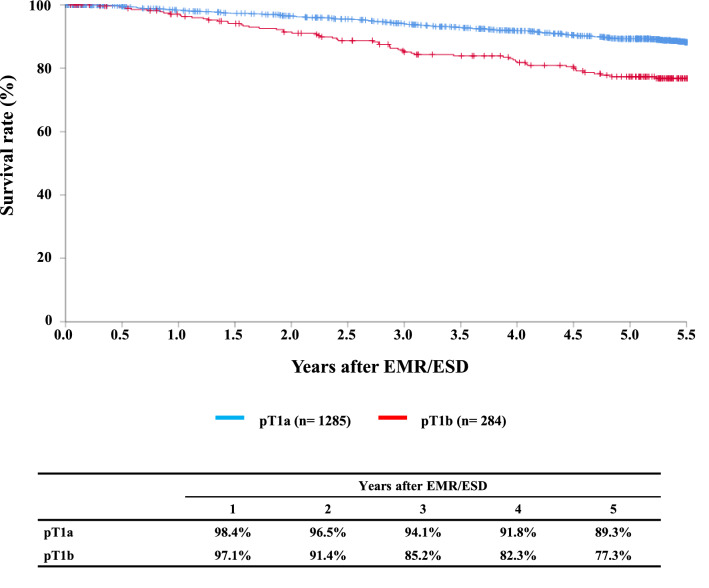
Survival of patients treated with EM/ESD according to the pathological depth of tumor invasion, pT (JES 10th)

#### Figure 3 Survival of patients treated with EMR/ESD according to the lymphatic and venous invasion

**Fig. 3:**
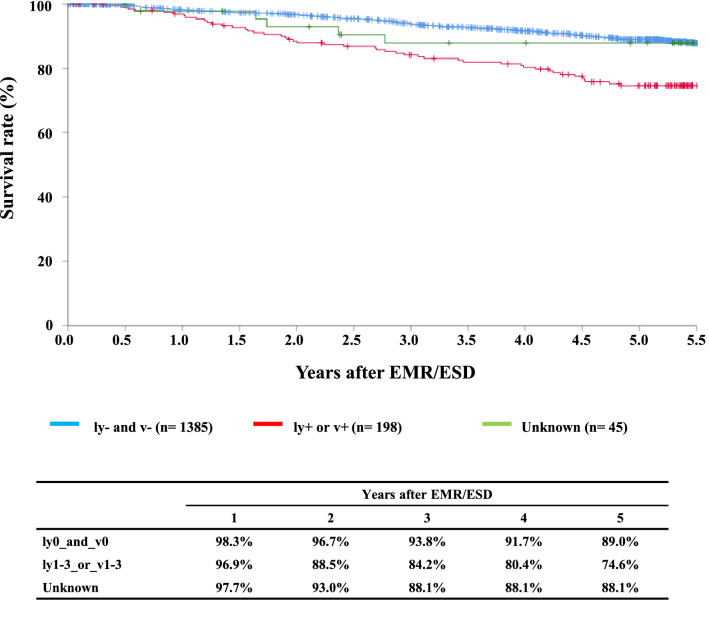
Survival of patients treated with EMR/ESD according to the lymphatic and venous invasion

### III. Results in patients treated with chemotherapy and/or radiotherapy in 2015

#### Table 12. Dose of irradiation (non-surgically treated cases)

**Table 12:** Dose of irradiation (non-surgically treated cases)

Dose of irradiation (Gy)	Definitive		Palliative (%)	Recurrence (%)	Others (%)	Total
	Radiation alone (%)	Chemoradiotherapy (%)				
-29	9 (4.3%)	13 (1.3%)	29 (9.3%)	0 (0.0%)	2 (28.6%)	53 (3.5%)
30–39	9 (4.3%)	12 (1.2%)	50 (16.0%)	3 (8.8%)	0 (0.0%)	74 (4.8%)
40–49	13 (6.3%)	32 (3.3%)	52 (16.6%)	5 (14.7%)	1 (14.3%)	103 (6.7%)
50–59	38 (18.3%)	260 (26.8%)	70 (22.4%)	11 (32.4%)	1 (14.3%)	380 (24.8%)
60–69	131 (63.0%)	619 (63.8%)	105 (33.5%)	15 (44.1%)	3 (42.9%)	873 (57.0%)
-70	7 (3.4%)	33 (3.4%)	6 (1.9%)	0 (0.0%)	0 (0.0%)	46 (3.0%)
Unknown	1 (0.5%)	1 (0.1%)	1 (0.3%)	0 (0.0%)	0 (0.0%)	3 (0.2%)
Total	208 (100.0%)	970 (100.0%)	313 (100.0%)	34 (100.0%)	7 (100.0%)	1532 (100.0%)
Median (min—max)	60.0 (1.8–92.0)	60.0 (1.8–99.0)	50.0 (1.8–99.0)	52.2 (30.0–66.0)	59.4 (14.0–61.2)	60.0 (1.8–99.0)

#### Table 13. Dose of irradiation (surgically treated cases)

**Table 13:** Dose of irradiation (surgically treated cases)

Dose of irradiation (Gy)	Preoperative irradiation (%)	Postoperative irradiation (%)
-29	6 (2.2%)	5 (8.1%)
30–39	30 (11.1%)	14 (22.6%)
40-49	194 (71.9%)	18 (29.0%)
50–59	23 (8.5%)	22 (35.5%)
60–69	17 (6.3%)	0 (0.0%)
-70	0 (0.0%)	0 (0.0%)
Unknown	1 (0.0%)	1 (1.6%)
Total Median	270	62
(min-max)	40.0 (20.0–66.0)	50.4 (1.8–61.2)

#### Figure 4 Survival of patients treated with chemotherapy and/or radiotherapy

**Fig. 4:**
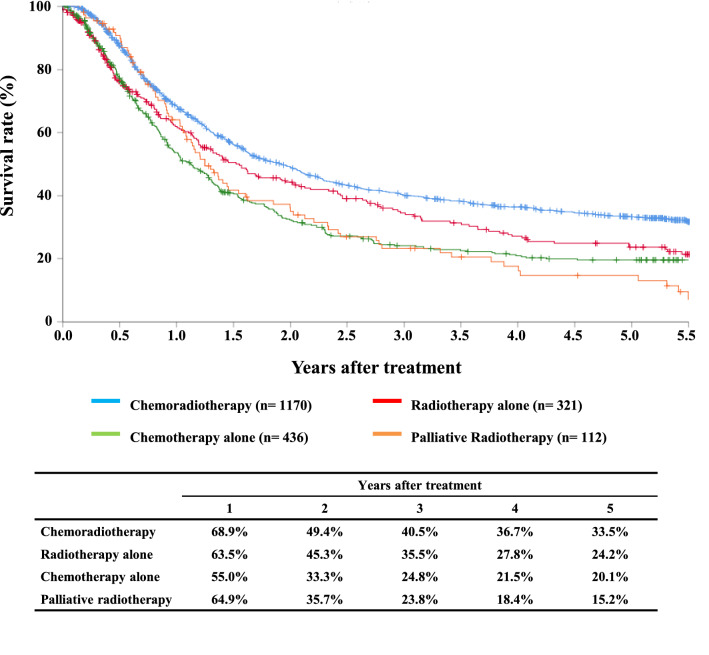
Survival of patients treated with chemotherapy and/or radiotherapy

#### Figure 5 Survival of patients treated with definitive chemoradiotherapy according to the clinical stage (UICC TNM 7th)

**Fig. 5:**
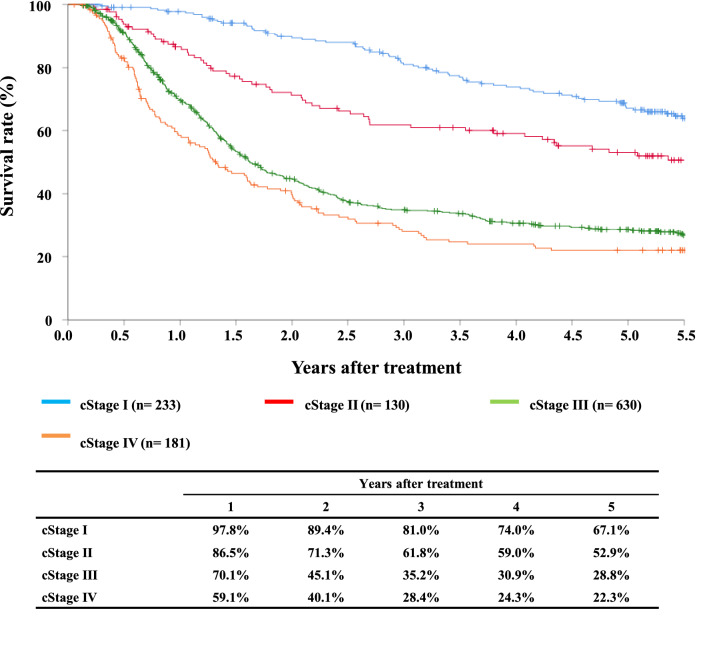
Survival of patients treated with definitive chemoradiotherapy according to the clinical stage (UICC TNM 7th)

#### Figure 6 Survival of patients who underwent radiotherapy alone according to the clinical stage (UICC TNM 7th)

**Fig. 6:**
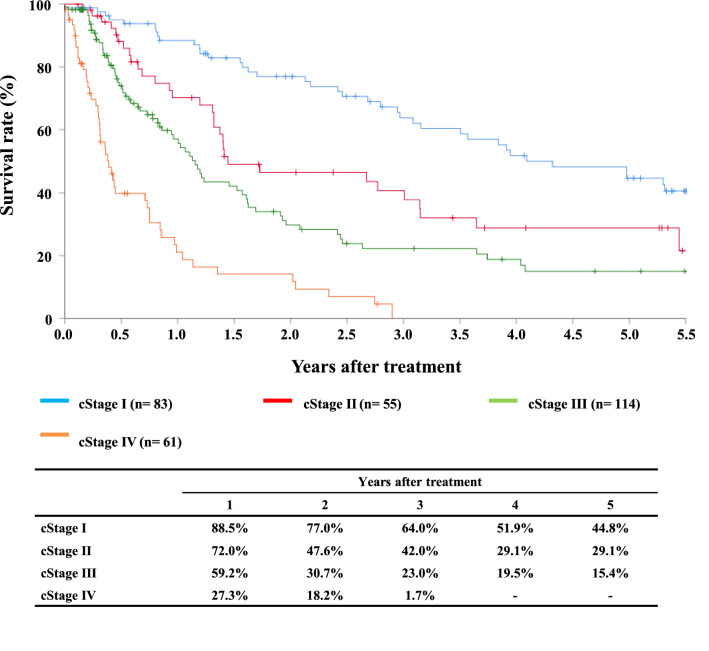
Survival of patients who underwent radiotherapy alone according to the clinical stage (UICC TNM 7th)

### IV. Results in patients who underwent esophagectomy in 2015

#### Table 14 Treatment modalities of esophagectomy

**Table 14:** Treatment modalities of esophagectomy

Treatment modalities	Cases (%)
Esophagectomy alone	2166 (41.9%)
Esophagectomy + postoperative chemotherapy	418 (8.1%)
Esophagectomy + postoperative chemoradiotherapy	109 (2.1%)
Esophagectomy + postoperative radiotherapy	30 (0.6%)
Preoperative chemotherapy + Esophagectomy	1901 (36.8%)
Preoperative chemoradiotherapy + Esophagectomy	266 (5.1%)
Definitive radiotherapy + Esophagectomy	7 (0.1%)
Definitive chemoradiotherapy + Esophagectomy	122 (2.4%)
Others	153 (3.0%)
Total	5172

#### Table 15 Tumor location

**Table 15:** Tumor location

Locations	Cases (%)
Cervical	181 (3.3%)
Upper thoracic	620 (11.3%)
Middle thoracic	2437 (44.5%)
Lower thoracic	1616 (29.5%)
EG	404 (7.4%)
E = G	106 (1.9%)
GE	93 (1.7%)
Unknown	22 (0.4%)
Total	5479

*EG* esophagogastric, *E* esophagus, *G* gastric

#### Table 16 Approaches to tumor resection

**Table 16:** Approaches to tumor resection

Approaches	Cases (%)
Cervical	143 (2.8%)
Right thoracic	4590 (88.7%)
Left thoracic	72 (1.4%)
Left thoracoabdominal	58 (1.1%)
Abdominal	133 (2.6%)
Transhiatal lower esophagectomy	71 (1.4%)
Transhiatal thoracic esophagectomy	81 (1.6%)
Sternotomy	9 (0.2%)
Others	9 (0.2%)
Unknown	6 (0.1%)
Total	5172

Thoracic includes thoracotomy and thoracoscopic

Abdominal includes laparotomy and laparoscopic

#### Table 17 Video-assisted surgery

**Table 17:** Video-assisted surgery

Video-assisted surgery	Cases (%)
None	2039 (39.4%)
Thoracoscopy	1480 (28.6%)
Thoracoscopy + Laparoscopy	1319 (25.5%)
Thoracoscopy + Laparoscopy + Mediastinoscopy	9 (0.2%)
Thoracoscopy + Mediastinoscopy	3 (0.1%)
Thoracoscopy + Other	2 (0.0%)
Laparoscopy	222 (4.3%)
Laparoscopy + Mediastinoscopy	16 (0.3%)
Laparoscopy + Mediastinoscopy + Other	0 (0.0%)
Mediastinoscopy	72 (1.4%)
Laparoscopy + Other	5 (0.1%)
Others	4 (0.1%)
Unknown	1 (0.0%)
Total	5172

#### Table 18 Fields of lymph node dissection according to the location of the tumor

**Table 18:** Fields of lymph node dissection according to the location of tumor

Field of lymphadenectomy	Cervical	Upper thoracic	Middle thoracic	Lower thoracic	Abdominal	E = G	GE	Unknown	Total
None	10 (6.3%)	9 (1.5%)	34 (1.5%)	36 (2.3%)	6 (1.6%)	0 (0.0%)	4 (5.2%)	1 (12.5%)	100 (1.9%)
C	31 (19.5%)	14 (2.4%)	31 (1.3%)	17 (1.1%)	1 (0.3%)	0 (0.0%)	0 (0.0%)	0 (0.0%)	94 (1.8%)
C + UM	19 (11.9%)	3 (0.5%)	5 (0.2%)	1 (0.1%)	2 (0.5%)	0 (0.0%)	0 (0.0%)	0 (0.0%)	30 (0.6%)
C + UM + MLM	8 (5.0%)	19 (3.2%)	48 (2.1%)	24 (1.5%)	1 (0.3%)	0 (0.0%)	1 (1.3%)	0 (0.0%)	101 (2.0%)
C + UM + MLM + A	73 (45.9%)	382 (65.3%)	1227 (53.2%)	626 (40.0%)	60 (16.0%)	11 (11.5%)	3 (3.9%)	4 (50.0%)	2386 (46.1%)
C + UM + A	1 (0.6%)	7 (1.2%)	15 (0.7%)	3 (0.2%)	1 (0.3%)	0 (0.0%)	0 (0.0%)	0 (0.0%)	27 (0.5%)
C + MLM	1 (0.6%)	1 (0.2%)	0 (0.0%)	2 (0.1%)	0 (0.0%)	0 (0.0%)	0 (0.0%)	0 (0.0%)	4 (0.1%)
C + MLM + A	1 (0.6%)	2 (0.3%)	13 (0.6%)	9 (0.6%)	2 (0.5%)	0 (0.0%)	0 (0.0%)	0 (0.0%)	27 (0.5%)
C + A	1 (0.6%)	0 (0.0%)	6 (0.3%)	4 (0.3%)	1 (0.3%)	0 (0.0%)	0 (0.0%)	0 (0.0%)	12 (0.2%)
UM	1 (0.6%)	2 (0.3%)	13 (0.6%)	7 (0.4%)	0 (0.0%)	0 (0.0%)	0 (0.0%)	0 (0.0%)	23 (0.4%)
UM + MLM	3 (1.9%)	12 (2.1%)	45 (2.0%)	22 (1.4%)	2 (0.5%)	0 (0.0%)	1 (1.3%)	0 (0.0%)	85 (1.6%)
UM + MLM + A	8 (5.0%)	116 (19.8%)	785 (34.0%)	667 (42.6%)	150 (39.9%)	39 (40.6%)	16 (20.8%)	2 (25.0%)	1783 (34.5%)
UM + A	1 (0.6%)	1 (0.2%)	4 (0.2%)	4 (0.3%)	1 (0.3%)	0 (0.0%)	0 (0.0%)	0 (0.0%)	11 (0.2%)
MLM	1 (0.6%)	4 (0.7%)	4 (0.2%)	11 (0.7%)	2 (0.5%)	2 (2.1%)	2 (2.6%)	0 (0.0%)	26 (0.5%)
MLM + A	0 (0.0%)	6 (1.0%)	62 (2.7%)	114 (7.3%)	109 (29.0%)	37 (38.5%)	29 (37.7%)	0 (0.0%)	357 (6.9%)
A	0 (0.0%)	7 (1.2%)	15 (0.7%)	17 (1.1%)	38 (10.1%)	7 (7.3%)	21 (27.3%)	1 (12.5%)	106 (2.0%)
Total	159	585	2,307	1,564	376	96	77	8	5172

*C* bilateral cervical nodes, *UM* upper mediastinal nodes, *MLM* middle-lower mediastinal nodes, *A* abdominal nodes

#### Table 19 Reconstruction route

**Table 19:** Reconstruction route

Route	Cases (%)
None	57 (1.1%)
Subcutaneous	319 (6.2%)
Retrosternal	2383 (46.1%)
Posterior mediastinal	1977 (38.2%)
Intrathoracic	317 (6.1%)
Cervical	67 (1.3%)
Others	44 (0.9%)
Unknown	8 (0.2%)
Total	5172

#### Table 20 Organs used for reconstruction

**Table 20:** Organs used for reconstruction

Organs	Cases (%)
None	69 (1.3%)
Whole stomach	212 (4.0%)
Gastric tube	4504 (85.4%)
Jejunum	210 (4.0%)
Free jejunum	85 (1.6%)
Colon	158 (3.0%)
Free colon	17 (0.3%)
Others	21 (0.4%)
Total organs	5,276
Total cases	5,103

#### Table 21 Histological classification

**Table 21:** Histological classification

Histological classification	Cases (%)
Squamous cell carcinoma	4,329 (83.7%)
Squamous cell carcinoma	925 (17.9%)
Well differentiated	727 (14.1%)
Moderately differentiated	2075 (40.1%)
Poorly differentiated	602 (11.6%)
Adenocarcinoma	316 (6.1%)
Barrett's carcinoma	139 (2.7%)
Adenosquamous carcinoma	34 (0.7%)
Mucoepidermoid carcinoma	2 (0.0%)
Basaloid carcinoma	87 (1.7%)
Neuroendocrine tumor	1 (0.0%)
Neuroendocrine carcinoma	29 (0.6%)
Undifferentiated carcinoma	8 (0.2%)
Malignant melanoma	22 (0.4%)
Carcinosarcoma	25 (0.5%)
GIST	3 (0.1%)
Adenoid cystic carcinoma	4 (0.1%)
Sarcoma	3 (0.1%)
Other carcinomas	7 (0.1%)
Other tumors	33 (0.6%)
Unknown	130 (2.5%)
Total	5,172

*GIST* gastrointestinal stromal tumor

#### Table 22 Pathological depth of tumor invasion, pT (JES 10th)

**Table 22:** Pathological depth of tumor invasion, pT (JES 10th)

Pathological depth of tumor invasion	Cases (%)
pT0	227 (4.4%)
pT1a	637 (12.3%)
pT1b	1470 (28.4%)
pT2	606 (11.7%)
pT3	1915 (37.0%)
pT4a	152 (2.9%)
pT4b	102 (2.0%)
pTX	63 (1.2%)
Total	5172

#### Table 23 Pathological grading of lymph node metastasis, pN (JES 10th)

**Table 23:** Pathological grading of lymph node metastasis, pN (JES 10th)

Lymph node metastasis	Cases (%)
pN0	2568 (49.7%)
pN1	926 (17.9%)
pN2	989 (19.1%)
pN3	349 (6.7%)
pN4	309 (6.0%)
Unknown	31 (0.6%)
Total	5172

#### Table 24 Pathological findings of lymph node metastasis, pN (UICC TNM 7th)

**Table 24:** Pathological grading of lymph node metastasis, pN (UICC TNM 7th)

Lymph node metastasis (Number of metastasis)	Cases (%)
pN0	2614 (50.5%)
pN1(1–2)	1353 (26.2%)
pN2(3–6)	754 (14.6%)
pN3(7-)	398 (7.7%)
pNX	53 (1.0%)
Total	5172

#### Table 25 Pathological findings of distant organ metastasis, pM (JES 10th)

**Table 25:** Pathological findings of distant organ metastasis, pM (JES 10th)

Distant metastasis (M)	Cases (%)
M0	5009 (96.8%)
M1	103 (2.0%)
Mx	60 (1.2%)
Total	5172

#### Table 26 Residual tumor

**Table 26:** Residual tumor

Residual tumor (R)	Cases (%)
R0	4667 (90.2%)
R1	241 (4.7%)
R2	152 (2.9%)
RX	112 (2.2%)
Total	5172

#### Table 27 Cause of death after esophagectomy

**Table 27:** Cause of death after esophagectomy

Cause of death	Cases (%)
Death due to recurrence	1809 (62.6%)
Death due to other cancer	205 (7.1%)
Death due to other diseases (with recurrence)	68 (2.4%)
Death due to other diseases (without recurrence)	404 (14.0%)
Death due to other diseases (recurrence unknown)	23 (0.8%)
Operative death*	41 (1.4%)
Postoperative hospital death**	77 (2.7%)
Unknown	264 (9.1%)
Total of death cases	2891

Follow-up period (months)	
Median (min–max)	59.76 (0.00–78.72)

*Operative death means death within 30 days after operation in or out of the hospital.

Operative mortality rate: 0.79%

**Hospital death is defined as death during the same hospitalization, regardless of department at the time of death. Hospital mortality rate: 2.3%

#### Figure 7 Survival of patients who underwent esophagectomy

**Fig. 7:**
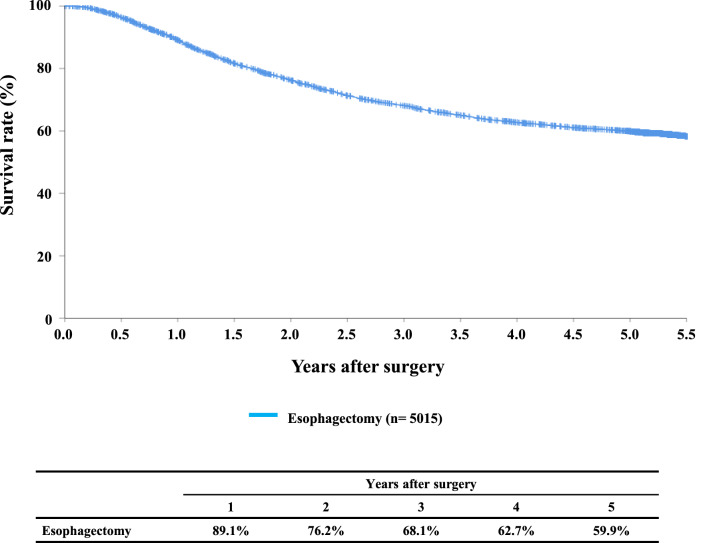
Survival of patients who underwent esophagectomy

#### Figure 8 Survival of patients who underwent esophagectomy according to the clinical stage (JES 10th)

**Fig. 8:**
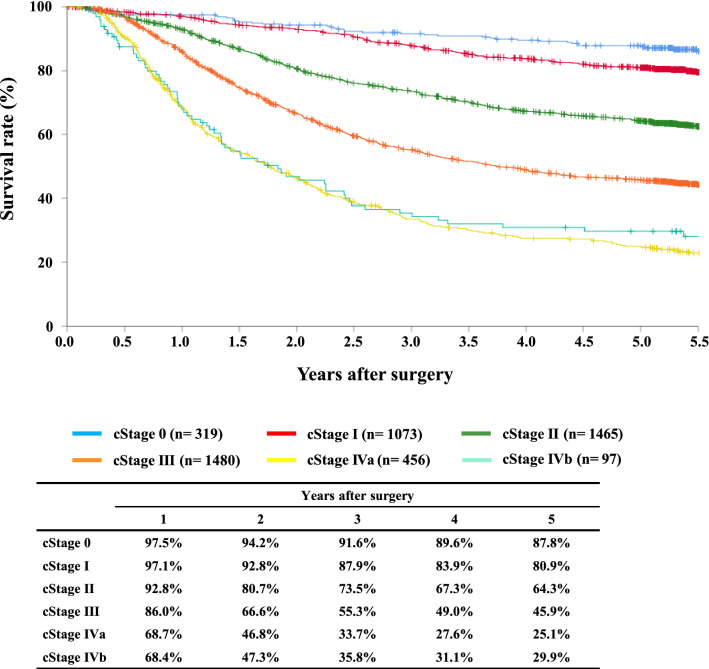
Survival of patients who underwent esophagectomy according to the clinical stage (JES 10th)

#### Figure 9 Survival of patients who underwent esophagectomy according to the clinical stage (UICC TNM 7th)

**Fig. 9:**
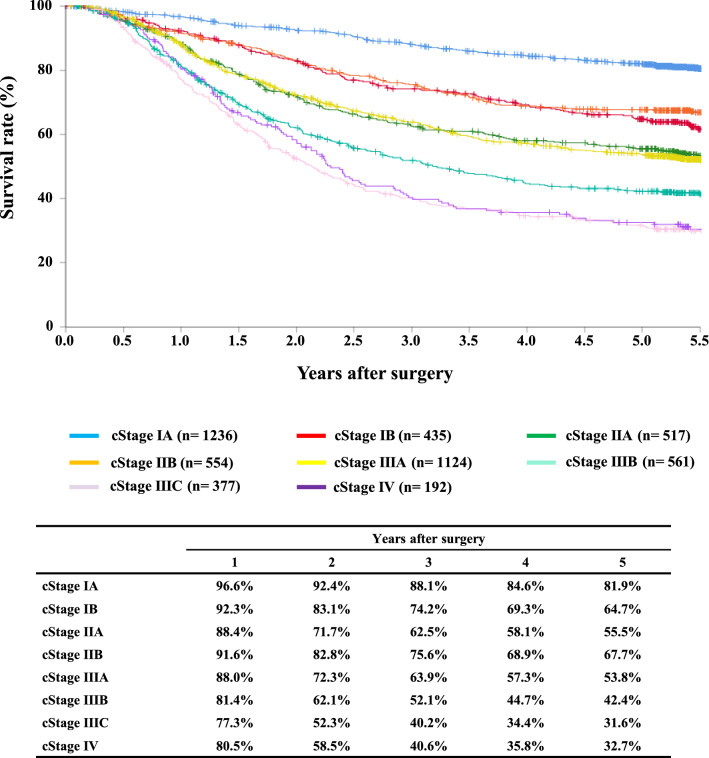
Survival of patients who underwent esophagectomy according to the clinical stage (UICC TNM 7th)

#### Figure 10 Survival of patients who underwent esophagectomy according to the depth of tumor invasion, pT (JES 10th)

**Fig. 10:**
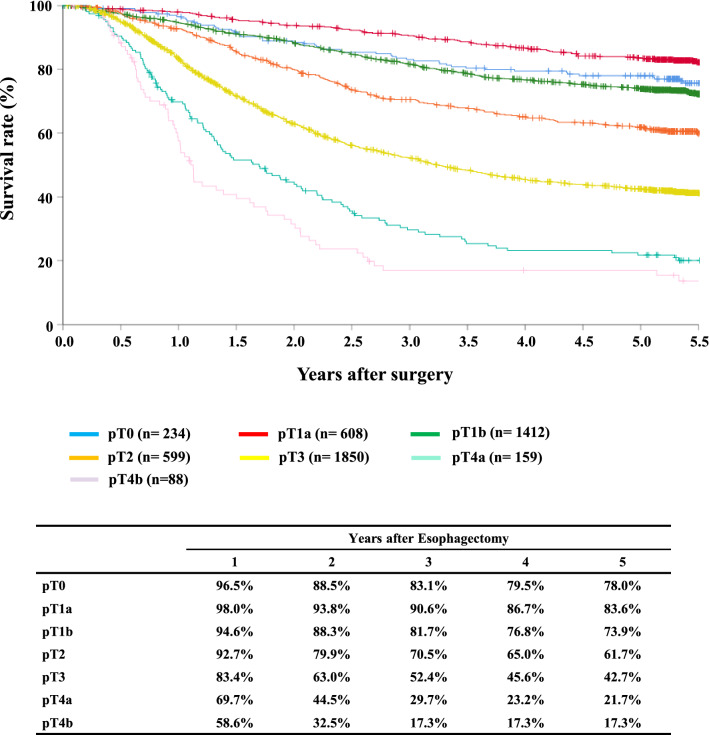
Survival of patients who underwent esophagectomy according to the depth of tumor invasion, pT (JES 10th)

#### Figure 11 Survival of patients who underwent esophagectomy according to lymph node metastasis (JES 10th)

**Fig. 11:**
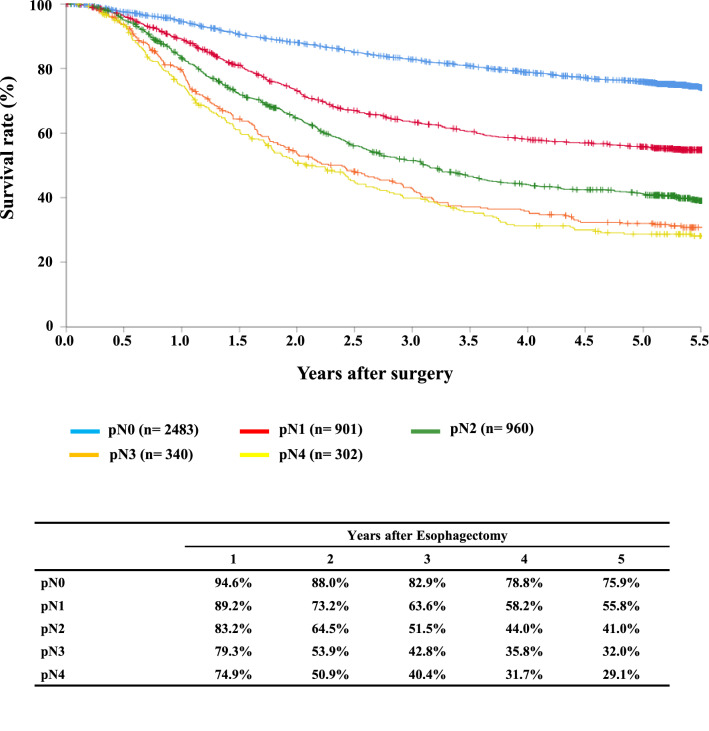
Survival of patients who underwent esophagectomy according to lymph-node metastasis (JES 10th)

#### Figure 12 Survival of patients who underwent esophagectomy according to lymph node metastasis (UICC TNM 7th)

**Fig. 12:**
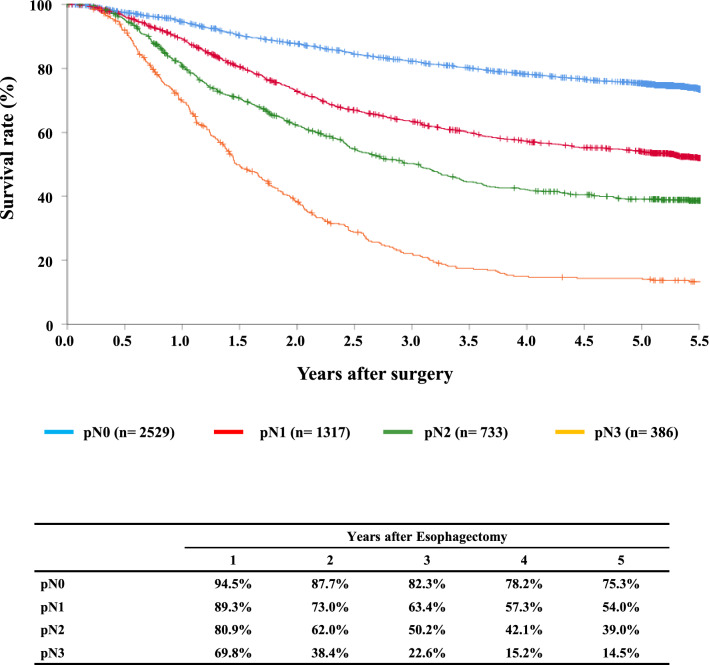
Survival of patients who underwent esophagectomy according to lymph-node metastasis (UICC TNM 7th)

#### Figure 13 Survival of patients who underwent esophagectomy according to the pathological stage (JES 10th)

**Fig. 13:**
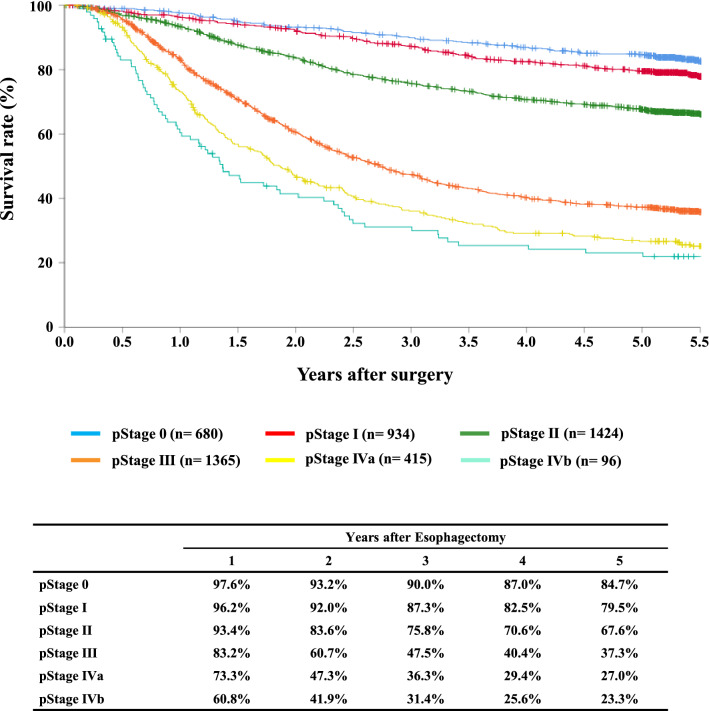
Survival of patients who underwent esophagectomy according to the pathological stage (JES 10th)

#### Figure 14 Survival of patients who underwent esophagectomy according to the pathological stage (UICC TNM 7th)

**Fig. 14:**
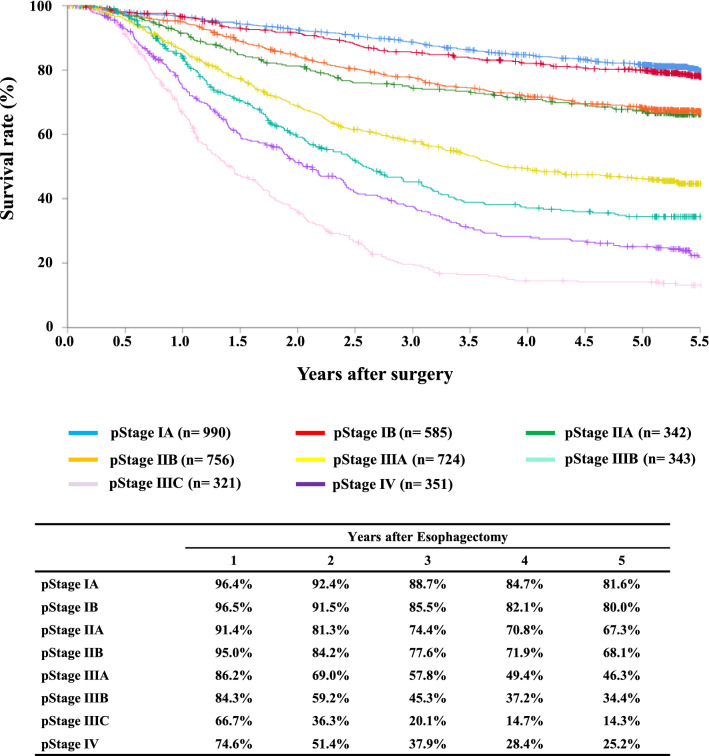
Survival of patients who underwent esophagectomy according to the pathological stage (UICC TNM 7th)

#### Figure 15 Survival of patients who underwent esophagectomy according to the residual tumor (R)

**Fig. 15:**
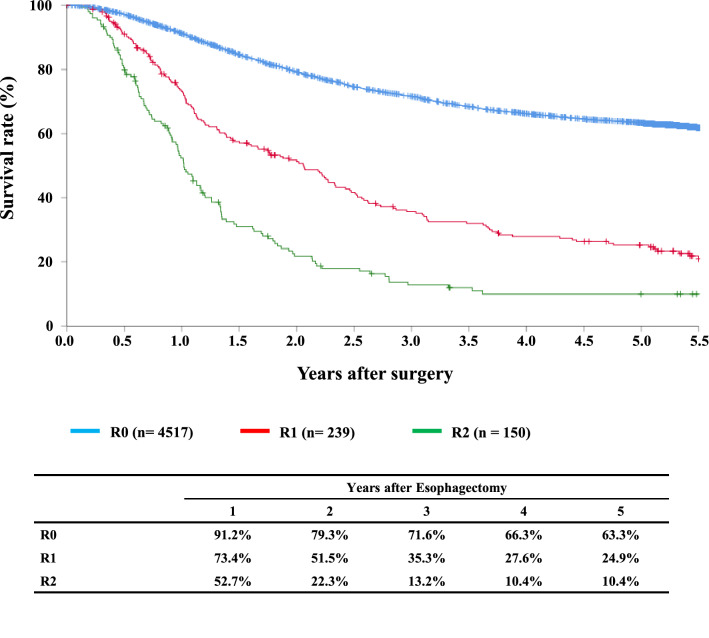
Survival of patients

## I. Clinical features of esophageal cancer patients treated in 2015

Institution-registered cases in 2015

**Table:** 

Institutions
Aomori Prefectural Central Hospital
Ageo Central General Hospital
Aichi Cancer Center
Aichi Medical University Hospital
Aizawa Hospital
Akita University Hospital
Arao Municipal Hospital
Asahi Rousai Hospital
Asahikawa Medical University Hospital
Cancer Institute Hospital of JFCR
Chiba Cancer Center
Chiba-Nishi General Hospital
Chiba University Hospital
Dokkyo Medical University Hospital
Dokkyo Medical University Saitama Medical Center
Edogawa Hospital
Ehime Prefectural Central Hospital
Eijyu General Hospital
Fuji City General Hospital
Fujioka General Hospital
Fujisaki Hospital
Fujisawa City Hospital
Fujita Health University Hospital
Fukaya Red Cross Hospital
Fukui University Hospital
Fukui-ken Saiseikai Hospital
Fukuoka City Hospital
Fukuoka Shin Mizumaki Hospital
Fukuoka University Chikushi Hospital
Fukuoka University Hospital
Fukuoka Wajiro Hospital
Fukushima Medical University Hospital
Fukushima Rosai Hospital
Fukuyama City Hospital
Gifu Prefectural General Center
Gifu Municipal Hospital
Gifu University Hospital
Gunma Prefectural Cancer Center
Gunma Saiseikai Maebashi Hospital
Gunma University Hospital
Hachinohe City Hospital
Hakodate City Hospital
Hakodate Goryokaku Hospital
Hakodate National Hospital
Hamamatsu University Hospital
Hamanomachi Hospital
Hannan Chuo Hospital
Hanyu General Hospital
Hasuda Hospital
Heartlife Hospital
Higashiosaka City Medical Center
Hiraka General Hospital
Hiratsuka City Hospital
Hirosaki University Hospital
Hiroshima City Asa Hospital
Hiroshima City Hospital
Hiroshima Memorial Hospital
Hiroshima Prefectural Hospital
Hiroshima Red Cross Hospital & Atomic-bomb Survivors Hospital
Hiroshima University Hospital
Hitachi General Hospital
Hofu Institute of Gastroenterology
Hokkaido University Hospital
Hospital of the University of Occupational and Environmental Health, Japan
Hyogo Cancer Center
Hyogo Prefectural Amagasaki General Medical Center
Hyogo Prefectural Nishinomiya Hospital
Ibaraki Prefectural Central Hospital
Iizuka Hospital
Ikeda City Hospital
Imari Arita Kyoritsu Hospital
International University of Health and Welfare Hospital
International University of Health and Welfare Mita Hospital
Iseikai Hospital
Ishikawa Prefectural Central Hospital
Itami City Hospital
Iwata City Hospital
Iwate Medical University Hospital
Iwate Prefectural Central Hospital
Iwate Prefectural Chubu Hospital
Iwate Prefectural Ofunato Hospital
JA Hiroshima General Hospital
JA Kouseiren Enshu Hospital
JA Onomichi General Hospital
Japanese Red Cross Ashikaga Hospital
Japanese Red Cross Fukuoka Hospital
Japanese Red Cross Ishinomaki Hospital
Japanese Red Cross Kitami Hospital
Japanese Red Cross Kyoto Daiichi Hospital
Japanese Red Cross Maebashi Hospital
Japanese Red Cross Medical Center
Japanese Red Cross Musashino Hospital
Japanese Red Cross Nagoya Daiichi Hospital
Japanese Red Cross Nagoya Daini Hospital
Japanese Red Cross Saitama Hospital
Japanese Red Cross Tottori Hospital
Japanese Red Cross Wakayama Medical Center
Japanese Red Cross Yamaguchi Hospital
JCHO Gunma Chuo Hospital
JCHO Kyushu Hospital
JCHO Osaka Hospital
Jichi Medical University Hospital
Jichi Medical University Saitama Medical Center
Juntendo University Hospital
Juntendo University Nerima Hospital
Juntendo University Shizuoka Hospital
Juntendo University Urayasu Hospital
Junwakai Memorial Hospital
Kagawa Prefectural Central Hospital
Kagawa Rosai Hospital
Kagawa University Hospital
Kagoshima City Hospital
Kagoshima Medical Association Hospital
Kagoshima University Hospital
Kaizuka City Hospital
Kakogawa Central City Hospital
Kanagawa Cancer Center
Kanagawa Prefectural Ashigarakami Hospital
Kanazawa Medical University Hospital
Kanazawa University Hospital
Kansai Denryoku Hospital
Kansai Medical University Hospital
Kansai Medical University Medical Center
Kansai Rosai Hospital
Kanto Central Hospital for Public School Teachers
Kashiwa Kousei General Hospital
Kawakita General Hospital
Kawasaki Medical School Hospital
Kawasaki Medical School Kawasaki Hospital
Kawasaki Municipal Hospital
Kawasaki Municipal Ida Hospital
Kawasaki Saiwai Hospital
Keio University Hospital
Keiyu Hospital
Keiyukai Sapporo Hospital
Kindai University Hospital
Kindai University Nara Hospital
Kinki Central Hospital
Kiryu Kousei General Hospital
Kishiwada City Hospital
Kitaharima Medical Center
Kitakyushu Municipal Medical Center
Kitano Hospital
Kitasato University Hospital
Kobe City Medical Center General Hospital
Kobe University Hospital
Kochi Health Science Center
Kochi University Hospital
Kohga Public Hospital
Kokura Memorial Hospital
Kosei Hospital
Kouseiren Takaoka Hospital
Kumagai General Hospital
Kumamoto University Hospital
Kumamoto Regional Medical Center
Kurashiki Central Hospital
Kurume University Hospital
Kyorin University Hospital
Kyoto Okamoto Memorial Hospital
Kyoto University Hospital
Kyoto-Katsura Hospital
Kyushu Central Hospital
Kyushu University Hospital
Matsudo City General Hospital
Matsushita Memorial Hospital
Matsuyama Red Cross Hospital
Mie University Hospital
Minamiosaka Hospital
Minoh City Hospital
Mito Red Cross Hospital
Mitsui Memorial Hospital
Miyazaki University Hospital
Moriguchi Keijinkai Hospital
Nagahama City Hospital
Nagahama Red Cross Hospital
Nagano Municipal Hospital
Nagaoka Chuo General Hospital
Nagasaki University Hospital
Nagoya City University Hospital
Nagoya City West Medical Center
Nagoya Tokushukai General Hospital
Nagoya University Hospital
Nanpuh Hospital
Nara City Hospital
Nara Medical University Hospital
National Cancer Center Hospital
National Cancer Center Hospital East
National Center for Global Health and Medicine
National Defence Medical College Hospital
Nerima Hikarigaoka Hospital
New Tokyo Hospital
NHO Beppu Medical Center
NHO Chiba Medical Center
NHO Disaster Medical Center
NHO Iwakuni Clinical Center
NHO Kanmon Medical Center
NHO Kure Medical Center
NHO Kyoto Medical Center
NHO Kyushu Cancer Center
NHO Kyushu Medical Center
NHO Matsumoto Medical Center
NHO Mito Medical Center
NHO Miyakonojo Medical Center
NHO Nagasaki Medical Center
NHO Oita Medical Center
NHO Osaka Medical Center
NHO Saitama Hospital
NHO Sendai Medical Center
NHO Shikoku Cancer Center
NHO Takasaki General Medical Center
NHO Tokyo Medical Center
NHO Yokohama Medical Center
Nihonkai General Hospital
Niigata Cancer Center Hospital
Niigata City General Hospital
Niigata Prefectural Central Hospital
Niigata Prefectural Shibata Hospital
Niigata University Medical & Detal Hospital
Nikko Memorial Hospital
Nippon Medical School Chiba Hokusou Hospital
Nippon Medical School Hospital
Nippon Medical School Musashi Kosugi Hospital
Nippon Medical School Tama Nagayama Hospital
Nishi Kobe Medical Center
Nissan Tamagawa Hospital
Northern Okinawa Medical Center
NTT Medical Center Tokyo
Numazu City Hospital
Obihiro Kousei Hospital
Ofuna Chuo Hospital
Ogaki Municipal Hospital
Ogikubo Hospital
Ogori Daiichi General Hospital
Ohara General Hospital
Ohta Hospital
Ohta Nishinouchi Hospital
Oita Prefectural Hospital
Oita Red Cross Hospital
Oita University Hospital
Okayama City Hospital
Okayama Red Cross General Hospital
Okayama Saiseikai General Hospital
Okayama University Hospital
Okitama Public General Hospital
Osaka City General Hospital
Osaka City University Hospital
Osaka General Medical Center
Osaka International Cancer Institute
Osaka Medical and Pharmaceutical University Hospital
Osaka Police Hospital
Osaka Red Cross Hospital
Osaka Rosai Hospital
Osaka University Hospital
Osaki City Hospital
Otsu City Hospital
Rinku General Medical Center
Saga Prefectural Hospital Koseikan
Saga University Hospital
Saiseikai Fukuoka General Hospital
Saiseikai Karatsu Hospital
Saiseikai Kawaguchi General Hospital
Saiseikai Noe Hospital
Saiseikai Utsunomiya Hospital
Saiseikai Yamaguchi General Hospital
Saiseikai Yokohama Tobu Hospital
Saitama Cancer Center
Saitama Citizens Medical Center
Saitama City Hospital
Saitama Medical University International Medical Center
Saitama Medical University Saitama Medical Center
Sakai City Medical Center
Saku Central Hospital
Seikei-kai Chiba Medical Center
Seirei Hamamatsu General Hospital
Sendai City Hospital
Sendai Kosei Hospital
Shiga General Hospital
Shiga University of Medical Science Hospital
Shimane Prefectural Central Hospital
Shimane University Hospital
Shin Takeo Hospital
Shinko Hospital
Shinshu University Hospital
Shizuoka Cancer Center
Shizuoka City Shizuoka Hospital
Shizuoka General Hospital
Showa University Hospital
Showa University Koto Toyosu Hospital
Southern Tohoku General Hospital
St. Luke's International Hospital
St. Marianna University School of Medicine Hospital
St. Mary's Hospital
Steel Memorial Yawata Hospital
Suita Municipal Hospital
Tachikawa Hospital
Takatsuki Red Cross Hospital
Tama Kyuryo Hospital
Teikyo University Chiba Medical Center
Teikyo University Hospital
Teikyo University Hospital Mizonokuchi
Teine Keijinkai Hospital
Tenri Hospital
The Hospital of Hyogo College of Medicine
The Jikei University Daisan Hospital
The Jikei University Hospital
Tochigi Cancer Center
Toda Central General Hospital
Toho University Ohashi Medical Center
Toho University Omori Medical Center
Toho University Sakura Medical Center
Tohoku University Hospital
Tokai University Hachioji Hospital
Tokai University Hospital
Tokai University Tokyo Hospital
Tokushima Red Cross Hospital
Tokushima University Hospital
Tokyo Dental College Ichikawa General Hospital
Tokyo Medical and Dental University Hospital
Tokyo Medical University Hachioji Medical Center
Tokyo Medical University Hospital
Tokyo Metropolitan Cancer and Infectious Diseases Center Komagome Hospital
Tokyo Metropolitan Tama Medical Center
Tokyo University Hospital
Tokyo Women's Medical University Hospital
Tokyo Women's Medical University Medical Center East
Tokyo Women's Medical University Yachiyo Medical Center
Tonan Hospital
Toranomon Hospital
Toshima Hospital
Tottori Prefectural Central Hospital
Tottori University Hospital
Toyama Prefectural Central Hospital
Toyama University Hospital
Toyonaka Municipal Hospital
Toyota Kosei Hospital
Toyota Memorial Hospital
Tsuchiura Kyodo Hospital
Tsukuba University Hospital
University Hospital, Kyoto Prefectural University of Medicine
University of the Ryukyus Hospital
Wakayama Medical University Hospital
Wakayama Rosai Hospital
Yamagata Prefectural Central Hospital
Yamagata University Hospital
Yamaguchi University Hospital
Yamanashi Prefectural Central Hospital
Yamanashi University Hospital
Yao Municipal Hospital
Yokkaichi Hospital
Yokohama City Minato Red Cross Hospital
Yokohama City Municipal Hospital
Yokohama City University Hospital
Yokohama City University Medical Center
Yokosuka General Hospital Uwamachi
Yuai Memorial Hospital

(Total 355 institutions).

## Patient background

Table 1, 2, 3, 4, 5, 6, 7, 8

## II. Results of endoscopically treated patients in 2015

Tables 9, 10, 11, and Figs. 1, 2, 3.

## III. Results in patients treated with chemotherapy and/or radiotherapy in 2015

Tables 12, 13 and Figs. 4, 5, 6.

## IV. Results in patients who underwent esophagectomy in 2015

Tables 14, 15, 16, 17, 18, 19, 20, 21, 22, 23, 24, 25, 26, 27, and Figs. 7, 8, 9, 10, 11, 12, 13, 14, 15
